# Association of Inpatient Use of Angiotensin-Converting Enzyme Inhibitors and Angiotensin II Receptor Blockers With Mortality Among Patients With Hypertension Hospitalized With COVID-19

**DOI:** 10.1161/CIRCRESAHA.120.317134

**Published:** 2020-06-02

**Authors:** Peng Zhang, Lihua Zhu, Jingjing Cai, Fang Lei, Juan-Juan Qin, Jing Xie, Ye-Mao Liu, Yan-Ci Zhao, Xuewei Huang, Lijin Lin, Meng Xia, Ming-Ming Chen, Xu Cheng, Xiao Zhang, Deliang Guo, Yuanyuan Peng, Yan-Xiao Ji, Jing Chen, Zhi-Gang She, Yibin Wang, Qingbo Xu, Renfu Tan, Haitao Wang, Jun Lin, Pengcheng Luo, Shouzhi Fu, Hongbin Cai, Ping Ye, Bing Xiao, Weiming Mao, Liming Liu, Youqin Yan, Mingyu Liu, Manhua Chen, Xiao-Jing Zhang, Xinghuan Wang, Rhian M. Touyz, Jiahong Xia, Bing-Hong Zhang, Xiaodong Huang, Yufeng Yuan, Rohit Loomba, Peter P. Liu, Hongliang Li

**Affiliations:** 1 From the Cardiology (P.Z., L.Z., J.-J.Q., J. Xie, Y.-M.L., Y.-C.Z., X. Huang, M.-M.C., X.C., Z.-G.S., X.-J.Z., H.L.), Renmin Hospital of Wuhan University; 2 Eye Center (X.Z.), Renmin Hospital of Wuhan University; 3 Neonatology (B.-H.Z.), Renmin Hospital of Wuhan University; 4 Medical Science Research Center (P.Z., Y.-X.J., H.L.), Zhongnan Hospital of Wuhan University; 5 Hepatobiliary and Pancreatic Surgery (D.G., H.W., Y. Yuan), Zhongnan Hospital of Wuhan University; 6 Cardiology (Y.P.), Zhongnan Hospital of Wuhan University; 7 Gastroenterology (J.L.), Zhongnan Hospital of Wuhan University; 8 Center for Evidence-Based and Translational Medicine (X.W.), Zhongnan Hospital of Wuhan University; 9 Urology (X.W.), Zhongnan Hospital of Wuhan University; 10 Institute of Model Animal of Wuhan University (P.Z., L.Z., F.L., J.-J.Q., Y.-M.L., Y.-C.Z., X. Huang, L. Lin, M.X., M.-M.C., X.C., Y.-X.J., J. Chen, Z.-G.S., X.-J.Z., H.L.); 11 Basic Medical School, Wuhan University (P.Z., L. Lin, H.L.); 12 Cardiology, The Third Xiangya Hospital, Central South University, Changsha, China (J. Cai); 13 Anesthesiology, Cardiovascular Research Laboratories, David Geffen School of Medicine, University of California, Los Angeles (Y.W.); 14 Centre for Clinic Pharmacology, The William Harvey Research Institute, Queen Mary University of London, United Kingdom (Q.X.); 15 Wuhan Kanghuashuhai Technology Company (R.T.), Wuhan; 16 Urology (P.L.), Wuhan Third Hospital & Tongren Hospital of Wuhan University; 17 Intensive Care Unit (S.F.), Wuhan Third Hospital & Tongren Hospital of Wuhan University; 18 Gastroenterology (X.H.), Wuhan Third Hospital & Tongren Hospital of Wuhan University; 19 Wuhan Ninth Hospital (H.C., M.L.); 20 Cardiology, The Central Hospital of Wuhan (P.Y., M.C.); 21 Cardiovascular Surgery, Union Hospital (J.Xia), Tongji Medical College, Huazhong University of Science and Technology; 22 Stomatology, Xiantao First People’s Hospital (B.X.); 23 General Surgery, Huanggang Central Hospital, Wuhan, China (W.M.); 24 General Surgery, Ezhou Central Hospital (L. Liu); 25 Infections Department, Wuhan Seventh Hospital (Y. Yan); 26 Institute of Cardiovascular and Medical Sciences, BHF Glasgow Cardiovascular Research Centre, University of Glasgow, United Kingdom (R.M.T.); 27 NAFLD Research Center, Division of Gastroenterology and Epidemiology, University of California San Diego, CA (R.L.); 28 Division of Cardiology, Department of Medicine, University of Ottawa Heart Institute, Ontario, Canada (P.P.L.).

**Keywords:** angiotensin-converting enzyme inhibitor, angiotensin II receptor blocker, coronavirus, COVID-19, hypertension, inpatients

## Abstract

Use of ACEIs (angiotensin-converting enzyme inhibitors) and ARBs (angiotensin II receptor blockers) is a major concern for clinicians treating coronavirus disease 2019 (COVID-19) in patients with hypertension.

**Objective:**

To determine the association between in-hospital use of ACEI/ARB and all-cause mortality in patients with hypertension and hospitalized due to COVID-19.

**Methods and Results:**

This retrospective, multi-center study included 1128 adult patients with hypertension diagnosed with COVID-19, including 188 taking ACEI/ARB (ACEI/ARB group; median age 64 [interquartile range, 55–68] years; 53.2% men) and 940 without using ACEI/ARB (non-ACEI/ARB group; median age 64 [interquartile range 57–69]; 53.5% men), who were admitted to 9 hospitals in Hubei Province, China from December 31, 2019 to February 20, 2020. In mixed-effect Cox model treating site as a random effect, after adjusting for age, gender, comorbidities, and in-hospital medications, the detected risk for all-cause mortality was lower in the ACEI/ARB group versus the non-ACEI/ARB group (adjusted hazard ratio, 0.42 [95% CI, 0.19–0.92]; *P*=0.03). In a propensity score-matched analysis followed by adjusting imbalanced variables in mixed-effect Cox model, the results consistently demonstrated lower risk of COVID-19 mortality in patients who received ACEI/ARB versus those who did not receive ACEI/ARB (adjusted hazard ratio, 0.37 [95% CI, 0.15–0.89]; *P*=0.03). Further subgroup propensity score-matched analysis indicated that, compared with use of other antihypertensive drugs, ACEI/ARB was also associated with decreased mortality (adjusted hazard ratio, 0.30 [95% CI, 0.12–0.70]; *P*=0.01) in patients with COVID-19 and coexisting hypertension.

**Conclusions:**

Among hospitalized patients with COVID-19 and coexisting hypertension, inpatient use of ACEI/ARB was associated with lower risk of all-cause mortality compared with ACEI/ARB nonusers. While study interpretation needs to consider the potential for residual confounders, it is unlikely that in-hospital use of ACEI/ARB was associated with an increased mortality risk.

## Abstract 2

Supplemental Digital Content is available in the text.

**In This Issue, see p 1667**

**Editorial, see p 1682**

The coronavirus disease 2019 (COVID-19) epidemic is caused by an infection with a novel coronavirus, officially named severe acute respiratory syndrome coronavirus 2 (SARS-COV-2).^1^ Among patients with COVID-19 admitted to a hospital, emerging data suggest that hypertension may be associated with an increased risk of mortality due to COVID-19.^2–4^

ACEIs (angiotensin-converting enzyme inhibitors) and ARBs (angiotensin II receptor blockers) are part of renin-angiotensin-aldosterone system (RAS) inhibiting agents and considered as one of the first-line medications for the management of a large proportion of patients with hypertension.^5,6^ However, continued use of ACEI/ARB has become controversial in the setting of COVID-19. The reason for this controversy stems from the fact that ACEIs and ARBs use may increase the expression of ACE2 receptor in animal-based studies,^7,8^ which is the known cellular receptor and a necessary entry point for SARS-COV-2 infection.^9^ Conversely, it has been indicated that ACE2 expression is downregulated following SARS infection, resulting in excessive activation of RAS and exacerbated pneumonia progression.^10^ Therefore, administration of ACEI/ARB may, in turn, be beneficial by blocking ACE2 downregulation-induced hyperactivation of RAS and thereby preventing acute lung injury. However, due to lack of sufficient clinical data supporting either the beneficial or harmful effects of ACEI/ARB use in patients with COVID-19, the optimal strategy for the management of hypertension in COVID-19 is uncertain and remains to be elucidated. The aim of this retrospective cohort study was to determine the association between in-hospital use of ACEI/ARB and all-cause mortality of COVID-19 among patients with hypertension.

## Methods

Materials and data that support the findings of this study are available from the corresponding authors upon reasonable request.

### Study Design and Participants

This retrospective, multi-center study included 1128 patients with hypertension and COVID-19 admitted to 9 hospitals in Hubei, China, including Renmin Hospital of Wuhan University, Zhongnan Hospital of Wuhan University, Wuhan First Hospital, Wuhan Third Hospital, Wuhan Seventh Hospital, Wuhan Ninth Hospital, Thunder Mountain Hospital, Huanggang Central Hospital, and the Central Hospital of Enshi Tujia and Miao Autonomous Prefecture. The study protocols were approved by central ethics committee, and all collaborating hospitals either approved study protocol by local ethics committees or accepted the central ethics approval. Patient informed consent was waived by each ethics committee. Patients were admitted between December 31, 2019 and February 20, 2020. The final date of follow-up was March 7, 2020.

COVID-19 was diagnosed by meeting one or both criteria of chest computerized tomography manifestations and reverse transcription-polymerase chain reaction according to the New Coronavirus Pneumonia Prevention and Control Program (fifth edition) published by the National Health Commission of China and World Health Organization interim guidance (Table I in the Data Supplement).^11,12^ We used the following inclusion and exclusion criteria to determine the study cohort. The inclusion criteria included patients with COVID-19, aged from 18 to 74 years, who were admitted to the above-mentioned hospitals in Hubei, China from December 31, 2019 to February 20, 2020. The exclusion criteria included incomplete medical records (eg, transfer to any other hospital), pregnancy, acute lethal organ injury (eg, acute myocardial infarction, acute coronary syndrome, acute pulmonary embolism, or acute stroke), decompensated or end stage of chronic organ dysfunction (eg, decompensated cirrhosis, decompensated chronic renal insufficiency, or severe congestive heart failure), AIDS, leukemia or malignancy. Patients with hypertension were classified based upon clearly documented medical history with systolic blood pressure ≥140 mm Hg or diastolic blood pressure ≥90 mm Hg.^5^

### Data Collection

Following data were collected including patient demographic information, medical history, clinical characteristics, laboratory data, radiological report data, history of comorbidities, therapeutic interventions during the hospitalization, and clinical outcomes. The patient demographic information (age and gender), clinical characteristics (fever, cough, fatigue, dyspnea, heart rate, respiratory rate, and blood pressure), and durations from symptom onset to admission were collected from electronic medical system. The radiological report data (chest computed tomography-diagnosed unilateral and bilateral lesions) were obtained from picture achieving and communication system. Laboratory data (blood cell count, CRP [C-reactive protein], procalcitonin, D-dimer, organ function markers, K^+^, LDL-c [low-density lipoprotein cholesterol], SpO2, and blood glucose) were collected from laboratory information system. Comorbidities (hypertension, diabetes mellitus, coronary heart disease, chronic renal diseases, cerebrovascular diseases, chronic liver disease, and chronic obstructive pulmonary disease) were extracted from medical history. The in-hospital medications and interventions were collected from doctor advices. Personal health identifying information (eg, name and ID) was anonymized, and each participant was given a study ID using an electronic coding system before data extraction to preserve patient privacy. Data were carefully reviewed and confirmed by experienced physicians and were double-checked to guarantee the accuracy of the data extraction procedures.

### Definition

The onset of COVID-19 was defined as the time point when the symptoms were first noticed. Patients with hypertension who received ACEI/ARB during hospitalization were classified as ACEI/ARB group. Patients with hypertension who did not receive ACEI/ARB during hospitalization were classified as non-ACEI/ARB group. In the subgroup propensity score-matched cohort analysis among patients taking antihypertensive medications, participants taking ACEI/ARB and other antihypertensive drugs (non-ACEI/ARB) during in-hospital stay were included. Patients discontinued treatment of hypertension due to inability to take medications (eg, hypotension, mechanical ventilation without nasal feeding and unable to orally taking medicines, or increase of creatinine level) during hospitalization were still included from the cohort. The primary end point was defined as 28-day all-cause death. Acute respiratory distress syndrome and septic shock were defined according to the World Health Organization interim guideline Clinical management of severe acute respiratory infection when novel coronavirus (2019-nCoV) infection is suspected^13^ (Table I in the Data Supplement). Acute kidney injury was defined as an elevation in serum creatinine level equal or above 26.5 μmmol/L within 48 hours.^14^ Cardiac injury was defined as the serum level of cTNI (cardiac troponin I), cTNT (cardiac troponin T), or hs-cTNI (high sensitivity cardiac troponin I) above the upper limit of normal. Disseminated intravascular coagulation was defined according to the criteria defined by the International Society on Thrombosis and Hemostasis.^15^ The increase in blood cell count and biochemical indexes were defined as over their upper limit of normal according to the criteria by the laboratory standards in each hospital (upper limits of normal for CRP, procalcitonin, creatinine, D-dimer, and platelets were shown in Table II in the Data Supplement).

### Propensity Score-Matched Analysis

Propensity score-matched cohorts were created based on variables which were expected to be potential confounders associated with exposure to ACEI/ARB, including age, gender, fever, cough, dyspnea, comorbidities (diabetes mellitus, coronary heart disease, and chronic renal disease), computerized tomography-diagnosed bilateral lung lesions, and incidence of increased CRP and creatinine. We adjusted for imbalanced variables (D-dimer, procalcitonin, and unilateral lesion) and in-hospital medications (antiviral drug and lipid-lowering drug) between ACEI/ARB versus non-ACEI/ARB groups in following mixed-effect Cox model. We used nonparametric missing value imputation, based on the missForest procedure in the R, to account for missing data on the laboratory variable of increased creatinine, CRP levels, procalcitonin, D-dimer, and unilateral lesion.^16^ A random forest model using the rest of the variables in the data set was performed to predict the missing values for these variables. The internally cross-validated errors were also estimated. ACEI/ARB and non-ACEI/ARB users were paired according to the propensity scores using exact matching with a caliper size of 0.05. The balance of covariates was evaluated by estimating SDs before and after matching, and small absolute value <0.1 was considered successful balancing between the 2 groups. For cohort analysis in all patients with hypertension, and in those treated with antihypertensive therapies, ACEI/ARB and non-ACEI/ARB ratios were paired at 1:2 and 1:1, respectively.

### Statistical Analysis

Continuous variables were expressed as median and interquartile range, and categorical variables were expressed as number and percentage (%). Statistical differences between 2 groups were analyzed using the Mann-Whitney *U* test for continuous variables, while categorical variables were compared using Fisher exact test or χ^2^ test. The risk of composite end points and corresponding hazard ratio (HR) were calculated using the Cox proportional hazard model comparing ACEI/ARB group versus non-ACEI/ARB group. Multi-variable adjusted including age, gender, comorbidities (diabetes mellitus, coronary heart disease, cerebrovascular disease, and chronic renal disease), and in-hospital medications (antiviral drug and lipid-lowering drug) were performed. We modeled site as a random effect in the mixed-effect Cox model. The proportional hazard assumptions were verified using correlation testing based on Schoenfeld residuals. Incidence rate differences (IRDs) were calculated to provide incidence difference on absolute change. The cumulative rates of death were compared using the Kaplan-Meier method. A 2-side α <0.05 was considered statistically different. Because of the potential for type 1 error due to multiple comparisons, findings for analyses of secondary end points should be interpreted as exploratory. Data were analyzed in R-3.6.3 (R Foundation for Statistical Computing, Vienna, Austria) and SPSS Statistics (version 23.0, IBM, Armonk, NY).

### Sensitivity Analysis

E-value analysis was conducted to assess the robustness of the association between ACEI/ARB use and all-cause mortality in the mixed-effect Cox model to address unmeasured confounding using the methodology of VanderWeele and Ding.^17–19^ The E-value is an alternative approach to sensitivity analyses for unmeasured confounding in our studies that avoids making assumptions that, in turn, require subjective assignment of inputs for some formulas. If the strength of unmeasured confounding is weaker than indicated by the E-value, then the main study result could not be overturned to one of the unmeasured confounder. E-values can, therefore, help to assess the robustness of the main study result by considering whether unmeasured confounding of this magnitude is plausible. We performed 2 sensitivity analyses to evaluate the robustness of propensity score-matched cohort analyses, among all patients with hypertension, using pairs of 1:2. In the first sensitivity analysis, comorbid diabetes mellitus was not included in matching, while the second sensitivity analysis was conducted adding cerebrovascular disease as a matching variable. We conducted a subset sensitivity analysis restricted to patients who were taking an antihypertensive medication, applying matching variables as above with the pairing ratio at 1:1.

## Results

### Participants

**Figure 1.**
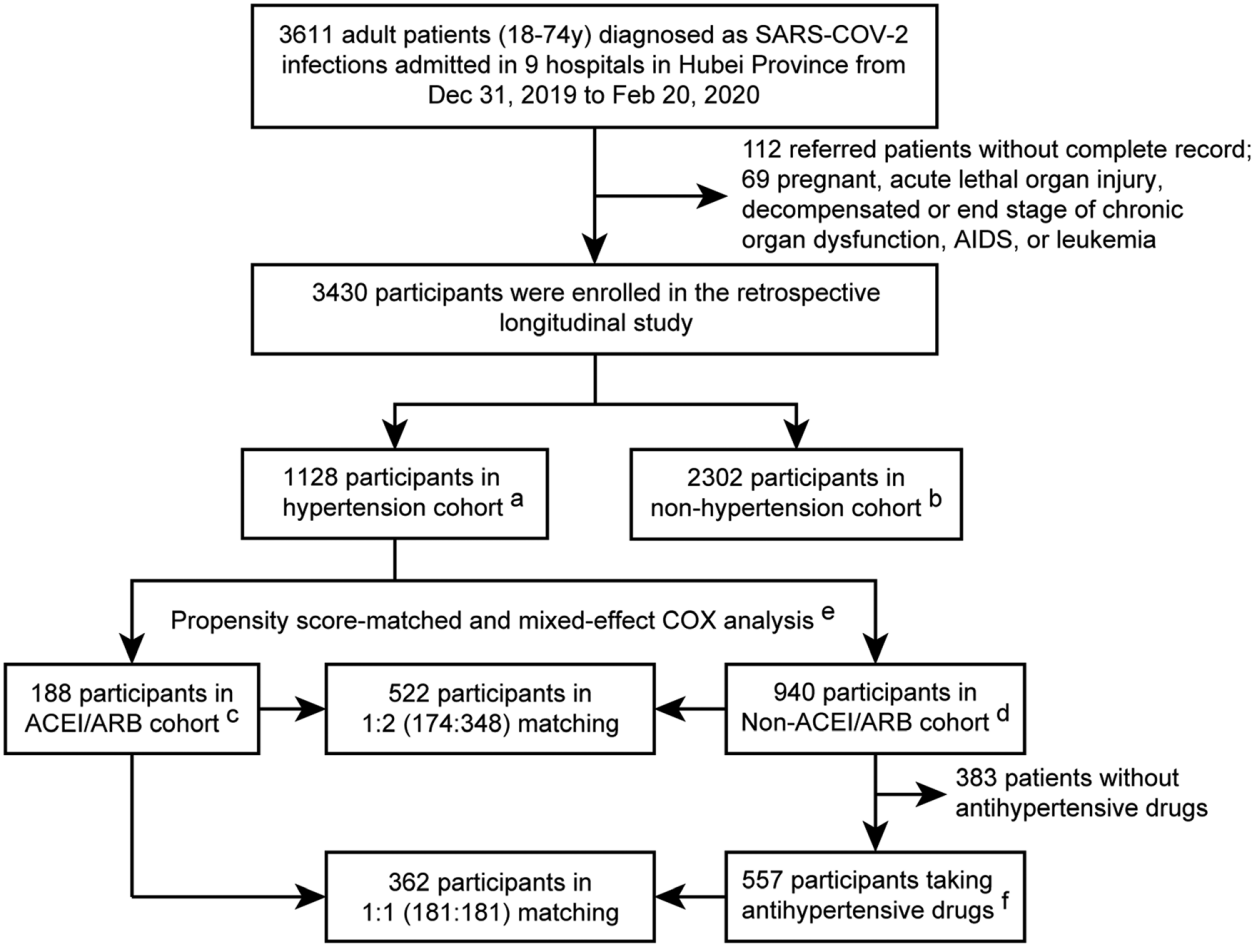
**The flowchart showing the strategy of participant enrollment**. ^a^, 1128 participants with a history of hypertension enrolled in the hypertension cohort. ^b^, 2302 participants without a history of hypertension enrolled in the nonhypertension cohort. ^c^, 188 patients with hypertension who taking ACEI (angiotensin-converting enzyme inhibitor) or ARB (angiotensin II receptor blocker) during hospitalization were enrolled in the ACEI/ARB cohort. Patients discontinued treatment of hypertension due to inability to take medications or hypotension were not excluded from the cohort. ^d^, 940 patients with hypertension who never taking ACEI and ARB during hospitalization were enrolled in the non-ACEI/ARB cohort. ^e^, Propensity score-matched age, gender, cough, dyspnea, comorbidities (diabetes mellitus, coronary heart disease, and chronic renal disease), chest computerized tomography (CT)-diagnosed lung lesions, and incidence of increased CRP (C-reactive protein) and creatine. Hospital site as a random effect in the mixed-effect Cox model. **^f^**, 557 patients with antihypertension drug who never taking ACEI and ARB during hospitalization were enrolled in the secondary non-ACEI/ARB cohort. SARS-COV-2 indicates severe acute respiratory syndrome coronavirus 2.

This study cohort included 3611 patients with COVID-19 who were admitted to these 9 hospitals in Hubei, China. After excluding 181 participants following our exclusion criteria, 3430 participants comprising 1128 hypertensive and 2302 normotensive cases were included in subsequent analysis (Figure 1; Table III in the Data Supplement). Among the 1128 participants with hypertension and COVID-19, 188 were classified as ACEI/ARB group (median age 64 [interquartile range, 55–68] years; 53.2% men) and the remaining 940 were classified as non-ACEI/ARB group (median age 64 [interquartile range, 57–69]; 53.5% men). The characteristics of the ACEI/ARB group versus the non-ACEI/ARB group on admission were provided in Table 1. Compared with the ACEI/ARB group, the non-ACEI/ARB group had higher prevalence of fever, dyspnea, and bilateral lung lesion at presentation. The dynamic changes in blood pressure during a 28-day follow-up period after presentation were similar between the ACEI/ARB and non-ACEI/ARB groups (Figure I in the Data Supplement). In terms of in-hospital treatment, the ACEI/ARB group had a higher percentage of patients receiving antiviral (88.8% versus 81.7%; *P*=0.02) and lipid-lowering therapies (22.9% versus 10.0%; *P*=1.51×10^−6^) than patients in the non-ACEI/ARB group (Table 2).

**Table 1.**
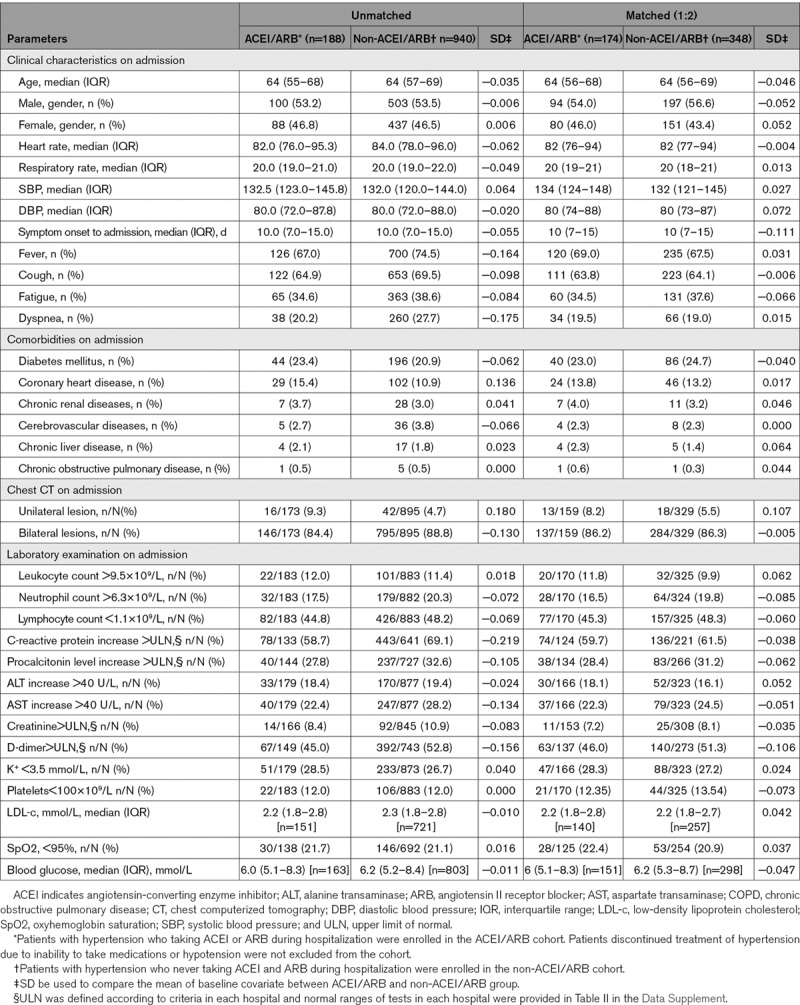
Characteristics of Patients With Hypertension in ACEI/ARB and Non-ACEI/ARB Groups Before and After Propensity Score Matching

**Table 2.**
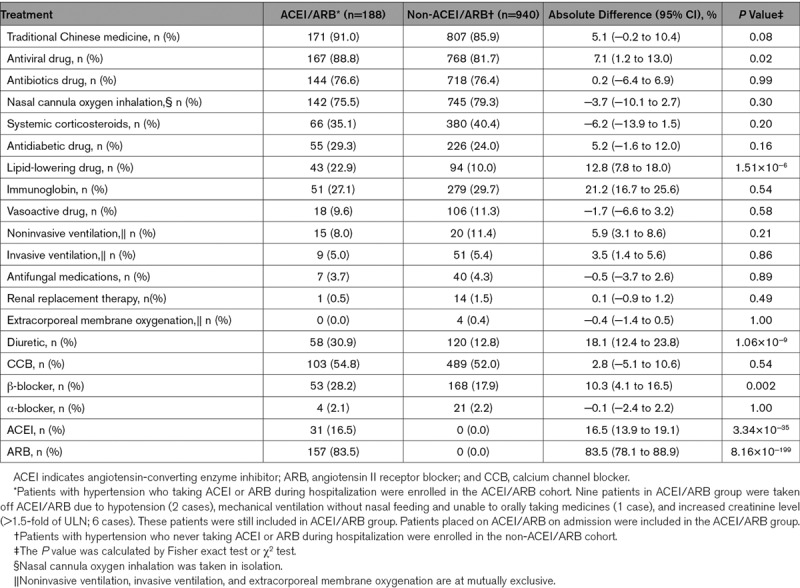
In-Hospital Management of ACEI/ARB and Non-ACEI/ARB Groups

### Primary Outcomes

**Figure 2.**
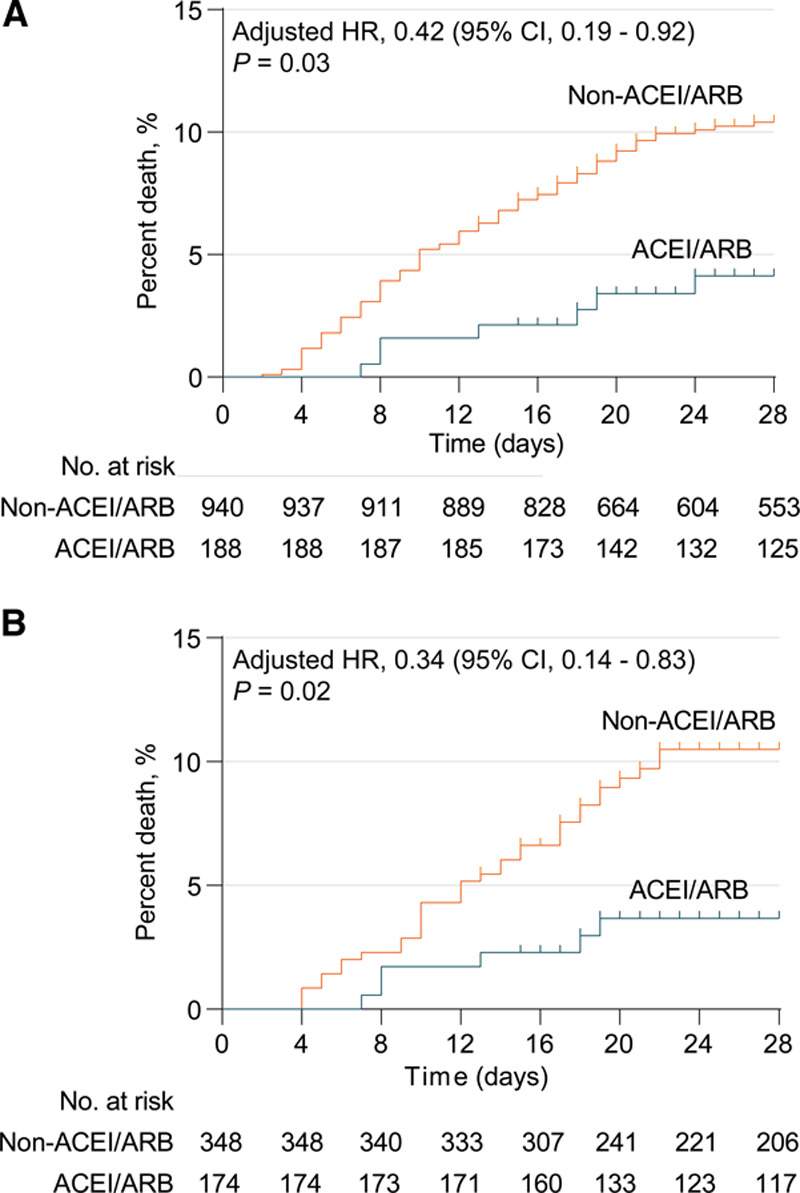
**Kaplan-Meier curves for cumulative probability of Coronavirus disease 2019 (COVID-19) mortality during 28-day follow-up duration in ACEI (angiotensin-converting enzyme inhibitor)/ARB (angiotensin II receptor blocker) or non-ACEI/ARB cohort among 1128 patients with hypertension.** The unmatched model and the median (interquartile range [IQR]) observation time was 28 (20–28) in ACEI/ARB cohort and 28 (19–28) in non-ACEI/ARB cohort (**A**). Propensity-score matched model and the median (IQR) observation time was 28 (20.5–28) in ACEI/ARB cohort and 28 (18–28) in non-ACEI/ARB cohort (**B**). The blips indicate censoring. HR indicates hazard ratio.

During a 28-day follow-up duration, 99 deaths died out of the 1128 patients with hypertension and COVID-19. The risk of 28-day all-cause mortality was significantly lower in ACEI/ARB group versus non-ACEI/ARB group (IRD, –0.24 [95%CI, –0.43 to –0.05]). In the mixed-effect Cox model using site as a random effect, after adjusting for age, gender, comorbidities, and in-hospital medications (antiviral and lipid-lowering drugs), use of ACEI/ARB was associated with lower all-cause mortality (adjusted HR, 0.42; 95% CI, 0.19-0.92; *P*=0.03) versus use of non-ACEI/ARB (Figure 2A and Table 3). The E value for the point estimate of primary end point was 4.22 with upper limit of CI at 1.41. In our study, the adjusted HR for association of known variables for all-cause mortality due to COVID-19 were 1.08 (95% CI, 1.04–1.11; *P*=3.30×10^−6^) for age, 2.23 (95% CI, 1.45−3.43; *P*=2.78×10^−4^) for gender, 1.47 (95% CI, 0.86–2.52; *P*=0.16) for coronary heart disease, and 1.35 (95% CI, 0.58–3.16; *P*=0.49) for cerebrovascular disease.

**Table 3.**
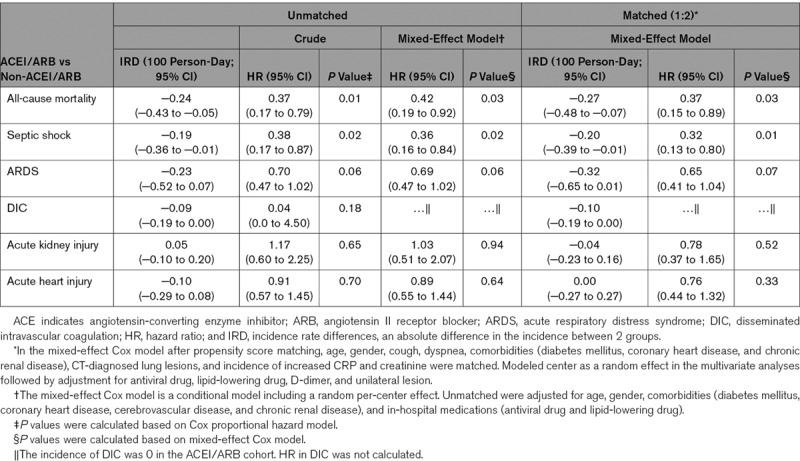
Hazard Ratios and Incidence Rate Ratios for Outcomes in ACEI/ARB Group vs Non-ACEI/ARB Group Under Mixed-Effect Cox Model and Propensity Score-Matching Model

We further conducted a propensity score-matched analysis to account for confounding that may have resulted in a protective association between ACEI/ARB use and all-cause mortality. We successfully matched 174 patients with hypertension in the ACEI/ARB group to non-ACEI/ARB group at a ratio of 1:2, and 522 individuals were included in the propensity score-matched samples. Imbalanced in-hospital medications (antiviral drug and lipid-lowering drug), increase of D-dimer, and unilateral lung lesion after matching were adjusted in mixed-effect Cox model using site as a random effect. The results remained consistent and statistically significant, demonstrating lower risk of all-cause mortality in patients who received ACEI/ARB (adjusted HR, 0.37 [95% CI, 0.15–0.89]; *P*=0.03) versus those who did not receive ACEI/ARB using this propensity score-matched analysis (Figure 2B and Table 3). To further assess the robustness of the association between ACEI/ARB use and mortality, we performed additional sensitivity analyses by using different matching variables. The results remained consistent and statistically significant in these sensitivity analyses with HRs of 0.34 (95% CI, 0.14–0.82; *P*=0.02) in the first sensitivity analysis and of 0.33 (95% CI, 0.13–0.80; *P*=0.01) in the second analysis.

Since there were 34.0% patients with hypertension who did not receive antihypertensive drugs during hospitalization, we performed subgroup propensity score-matching analysis on the remaining 745 patients who received at least one antihypertensive medication during hospitalization to further minimize the potential bias from nonusers. One hundred eighty-one patients using ACEI/ARB versus those using other antihypertensive drugs were paired at 1:1. Characteristics of matched and unmatched participants in this analysis were listed in Table IV in the Data Supplement. The results demonstrated that the in-hospital use of ACEI/ARB was associated with lower risk of all-cause mortality (adjusted HR, 0.29 [95% CI, 0.12–0.69]; *P*=0.005) due to COVID-19 (Figure II and Table V in the Data Supplement). This association was further supported by sensitivity analyses with adjusted HR of 0.29 (95% CI, 0.12–0.70; *P*=0.01) in the first sensitivity analysis not including diabetes mellitus as a matching variable and of 0.30 (95% CI, 0.13–0.71; *P*=0.01) in the second sensitivity analysis adding cerebrovascular disease as a matching variable.

### Secondary Outcomes

The incidence of septic shock (IRD, −0.19 [95% CI, −0.36 to −0.01]) and disseminated intravascular coagulation (IRD, −0.09 [95%CI, −0.09 to 0.00]) were lower in the ACEI/ARB group than the non-ACEI/ARB group (Table 3). In the mixed-effect Cox model, use of ACEI/ARB was associated with lower risk of septic shock (adjusted HR, 0.36 [95% CI, 0.16–0.84]; *P*=0.02) compared with non-ACEI/ARB group (Table 3).

In propensity score-matched cohort analysis, the risk of septic shock was lower in ACEI/ARB group (adjusted HR, 0.32 [95% CI, 0.13−0.80]; *P*=0.01; IRD, −0.20 [95% CI, −0.39 to −0.01]) than non-ACEI/ARB group among all individuals with hypertension (Table 3). In a sub-group of patients, who received at least one antihypertensive medication during hospitalization, the findings remained consistent (adjusted HR, 0.24 [95% CI, 0.10−0.63], *P*=0.003; IRD, −0.31 [95% CI, −0.54 to −0.09] for septic shock; Table V in the Data Supplement).

### Comparison of Patients With Hypertension Versus Nonhypertension

Compared with patients in the normotensive group, the hypertensive group had a higher percentage of dyspnea, comorbidities, and abnormal laboratory markers on admission, and they received more intensive in-hospital interventions (Table III and Table VI in the Data Supplement). During the 28-day follow-up period, co-existing hypertension was significantly associated with the higher risk of all-cause mortality (adjusted HR, 1.41 [95% CI, 1.03−1.94]; *P*=0.03) and the occurrence of multi-organ injury (Figure III and Table VII in the Data Supplement).

## Discussion

In this multicenter retrospective study, in-hospital use of ACEI/ARB was associated with lower risk of all-cause mortality due to COVID-19 compared with either nonuse of ACEI/ARB or use of a different class of antihypertensive agent among patients with hypertension. Although it is plausible that unmeasured confounding may have contributed to the observed protective association, these data suggest that in-hospital use of ACEI/ARB was not associated with increased mortality in COVID-19. These findings provide clinical evidence in support of recently published guidance statements by several international societies to continue ACEI/ARB in patients with COVID-19.^20,21^ Given the retrospective nature of this study, these data need further validation in geographically diverse, prospective, cohort studies. Randomized controlled trials are needed to examine the efficacy of ACEI/ARB use in patients with hypertension and COVID-19.

Previous clinical studies have shown that hypertension is a risk factor for higher mortality in patients suffering from SARS and Middle East Respiratory Syndrome.^22–24^ Similar to the association between hypertension and other previous outbreaks from coronavirus infections, recent studies in patients with COVID-19 have also reported that hypertension is associated with higher mortality in COVID-19 compared with normotensions.^3,4^ The underlying pathogenic mechanism linking hypertension and severity of COVID-19 infection remains to be elucidated. It has been hypothesized that excessive activation of RAS system might contribute to progression of COVID-19 related lung injury by promoting inflammatory response and cytokine storm,^25^ stimulating the NADH/NADPH oxidase system,^26^ and triggering cell contraction and vasoconstriction.^27^ Further studies are needed to better understand the underlying biological mechanisms involved in the association between hypertension and adverse outcomes in COVID-19.

In the management of patients with COVID-19 with hypertension, use of ACEI or ARB has been a contentious issue. These therapeutic effects involve ACE2, a known cellular receptor of SARS-COV-2 which is required for viral entry and propagation in host cells. ACEI and ARB treatment can increase ACE2 expression in animal-based studies.^7,8^ Potentially, ACEI/ARB might increase ACE2 expression, thus promoting SARS-COV-2 susceptibility and disease severity of COVID-19. Conversely, ACE2 negatively regulates RAS and serves as a counterbalance to ACE function. ACE2 expression is significantly downregulated after SARS-COV infection, contributing to hyper-activated RAS cascades.^28^ As a consequence, loss of ACE2 in mice confers resistance to SARS-COV infection, but also results in exacerbated vascular permeability, lung edema, neutrophil accumulation, and pulmonary dysfunction.^10,29^ Recombinant ACE2 protein protects against acute lung injury in mouse models of acute respiratory distress syndrome and SARS. A retrospective review of 539 consecutive hospitalized patients with viral pneumonia indicates that continuing in-hospital use of ACEI or ARB may reduce the risk of pneumonia and death (ACEI, odds ratio, 0.64 for risk of pneumonia; odds ratio, 0.25 for in-hospital death; ARB, odds ratio, 0.48 for risk of pneumonia; odds ratio, 0.75 for in-hospital death).^30^ A more recent study by Liu et al shows that plasma Ang II concentration is significantly elevated after SARS-COV-2 infection.^31^ The influence of ACEI/ARB on COVID-19 related outcomes has not been fully investigated. Furthermore, among patients with chronic obstructive pulmonary disease, it has been suggested that ARB might be more effective than ACEI to reduce the severity and mortality due to chronic obstructive pulmonary disease.^32^ This study was not powered to assess the differential beneficial effects of ACEI versus ARB in improving outcomes in COVID-19. The differential efficacy of ACEI and ARB use in improving COVID related outcomes also needs to be examined in further studies.

A statement jointly published by American Heart Association, the Heart Failure Society of America and the American College of Cardiology,^20^ and another statement from the International Society of Hypertension on COVID-19^21^ strongly recommended continuation of ACEI or ARB among patients with co-existing hypertension and COVID-19. The main argument in favor of continuation of ACEI/ARB was that there are no clinical data to show whether ACEI and ARB would either improve or worsen COVID-19. Therefore, it is not recommended to modify or change antihypertensive therapy before fully evaluating the possible influence of ACEI/ARB among patients with COVID-19. Our findings in this article provide evidence supporting continuous use of ACEI/ARB for patients with hypertension infected with SARS-COV-2. Comorbidities and in-hospital medications might lead confounding to the association between ACEI/ARB application with COVID-19 mortality. Previous studies have suggested that hypokalemia may be a marker of unopposed Ang II,^33,34^ and therefore, hypokalemia may modify the association between anti-hypertensive therapy and outcomes in COVID-19 infection. Further studies are needed to examine the role of hypokalemia and hypertension and ACEI/ARB use and its influence on COVID-19 severity. The protection effect of ACEI/ARB also may result from the favorable effect on microvascular complications, thus reduces cardiovascular and renal morbidity.^35,36^

Overall, these findings suggested potential beneficial effects observed with continued use of ACEI/ARB therapy and the possible contribution of RAS activation in the pathogenesis of severity of COVID-19 in patients with hypertension. Considering the impact of unmeasured potential confounders, we conducted E-value analysis and found that E-value was substantially greater than accepted risk factors for COVID-19 mortality. Therefore, it is not likely that an unmeasured confounder exists to modify the conclusion that ACEI/ARB use was not associated with increased COVID-19 mortality as observed in this study.

### Limitations

This study has several limitations. First, the study population originated from 9 hospitals in Hubei Province and may reflect natural history of hospitalized patients with COVID-19. It is plausible that the beneficial effects of ACEI/ARB may be different among patients who are managed in the outpatient setting or in ethnically or geographically diverse populations. Second, the study sample-size was modest and included 188 patients who received ACEI/ARB. It did not have the power to detect if there was a differential effect between ACEI and ARB. Third, since the retrospective nature of this study, some parameters were not available in all patients, and in-hospital medications might be not fully recorded. For instance, some patients with hypertension may have failed to report antihypertensive medication though self-administration (eg, Traditional Chinese Medicines) or might stop antihypertensive medication due to well-controlled blood pressure. Fourth, application of antihypertensive drugs was not matched or adjusted when comparing ACEI/ARB and non-ACEI/ARB groups since it is a key comparative factor for outcomes. The differences in proportions of patients using β-blocker and diuretics between ACEI/ARB and non-ACEI/ARB groups might induce unappreciated confounding to conclusion. Fifth, this study was not able to retrieve prehospital self-medications from in-hospital electronic record systems in the urgent circumstance of the COVID-19 pandemic, and thus was unable to account for their influence on the severity of presentation and outcomes. Therefore, large-scale prospective cohort studies and randomized controlled trials are needed in ethnically and geographically diverse cohorts to better understand the association between ACEI/ARB and survival in COVID-19.

### Conclusions

Among patients with hypertension hospitalized with COVID-19, inpatient treatment with ACEI/ARB was associated with lower risk of all-cause mortality compared with ACEI/ARB nonusers. While study interpretation needs to consider the potential for residual confounders, it is unlikely that inpatient ACEI/ARB would be associated with an increased risk of mortality.

## Author Contributions

P. Zhang, L. Zhu, F. Lei, J. Cai, and J.J. Qin designed study, collected and analyzed data, and wrote article. Y.-C. Zhao, X. Huang, L. Lin, M. Xia, M.-M. Chen, Y.-X. Ji, H. Wang, J. Lin, P. Luo, S. Fu, H. Cai, P. Ye, B. Xiao, W. Mao, L. Liu, Y. Yan, M. Liu, M. Chen, B.-H. Zhang, X. Wang, J. Xia, and X. Huang collected and reviewed clinical, laboratory, and radiological data. Y.-M. Liu, X. Cheng, and J. Chen performed statistical analysis. J. Xie, X. Zhang, D. Guo, and Y. Peng reviewed, interpreted, and checked clinical data. X.-J. Zhang, Z.-G. She, Y. Wang, Q. Xu, R. Tan, and R.M. Touyz wrote article and provided valuable suggestions for study design and data analysis. Y. Yuan, R. Loomba, P. Luo, and H. Li contributed equally, designed the project, edited article, and supervised the study. All authors have approved the final version of this article.

## Sources of Funding

This work was supported by grants from National Key R&D Program of China (2016YFF0101504, 2020YFC0845500), the National Science Foundation of China (81630011, 81970364, 81970070, 81970011, 81870171, and 81700356), the Major Research Plan of the National Natural Science Foundation of China (91639304), the Hubei Science and Technology Support Project (2019BFC582, 2018BEC473, and 2017BEC001), and Medical flight plan of Wuhan University.

## Disclosures

None.

Supplemental Materials

Online Tables I–VII

Online Figures I–III

## Supplementary Material



## Novelty and Significance

### What Is Known?

- Hypertension is the most common comorbidity of the coronavirus disease 2019 (COVID-19) and had been suggested to be associated with increased mortality.
- ACEIs (angiotensin-converting enzyme inhibitors) and ARBs (angiotensin II receptor blockers) are first-line medications for a large proportion of patients with hypertension.
- Use of ACEI/ARB is a major concern for clinicians in treating COVID-19 patients with hypertension because of the potential effect of ACEI/ARB on increasing the expression of ACE2 (angiotensin-converting enzyme 2), the binding receptor and entry point of the SARS-COV-2.
