# Supplementary material for: Association of Inpatient Use of Angiotensin-Converting Enzyme Inhibitors and Angiotensin II Receptor Blockers With Mortality Among Patients With Hypertension Hospitalized With COVID-19
Source: Circ Res. 2020 Jun 2;126(12):1671–81. doi: 10.1161/CIRCRESAHA.120.317134 (PMC7265882; doi:10.1161/CIRCRESAHA.120.317134)
Supplement: Supplementary file 1 [file res-126-1671-s001.pdf]

## Supplemental Materials

P Zhang, L Zhu, J Cai *et al.* Association of inpatient use of angiotensin converting enzyme inhibitors and angiotensin II receptor blockers with mortality among patients with hypertension hospitalized with COVID-19

**Online Table I**. Diagnostic criteria of COVID-19 (Exclusive for patients from Hubei Province) and septic shock in the study

**Online Table II.** The normal range of serum CRP, procalcitonin, creatinine, D-dimer, and platelet test in study hospitals

**Online Table III**. Clinical characteristics, laboratory examination and CT features of patients on admission

**Online Table IV**. Characteristics of ACEI/ARB and non-ACEI/ARB groups among patients with hypertension taking antihypertensive drugs before and after propensity score-matching

**Online Table V** Hazard ratio and incidence rate ratios for association between ACEI/ARB and COVID-19 outcomes among patients with hypertension taking antihypertensive drugs in mixed-effect Cox model after propensity score-matching

**Online Table VI**. In-hospital management of participants

**Online Table VII.** Hazard Ratio for primary and secondary outcome in hypertension and non-hypertension group

**Online Figure I** Dynamic change of blood pressure and mean arterial pressure of patients with hypertension treated with ACEI/ARB or without ACEI/ARB.

**Online Figure II.** Kaplan-Meier Curves for cumulative probability of COVID-19 mortality among individuals with inpatient use of ACEI/ARB (ACEI/ARB cohort) or taking other antihypertensive drugs (non-ACEI/ARB cohort) in propensity score-matched analysis.

**Online Figure III.** The 28-day Kaplan-Meier Curves of hypertensive and non-hypertensive cohort.

This supplementary material has been provided by the authors to give readers additional information about their work.

**Online Table I** Diagnostic criteria of COVID-19 (Exclusive for patients from Hubei Province) and septic shock in the study

| | |
|---|---|
| **COVID-19 diagnosis**: Any a positive result on below, irrespective of clinical signs and symptoms | |
| (1) Typical CT manifestations of viral pneumonia | Primary reported findings on CT: 1. bilateral, subpleural or peripheral ground-glass opacities; 2. lobular septal thickening; 3. air space consolidation; 4. bronchovascular thickening in the lesion; 5.traction bronchiectasis |
| (2) RT-PCR* for COVID-19 nasal and pharyngeal swab specimens or blood sample | + |
| **Septic shock definition**: Any a positive result on below | |
| (1) Persisting hypotension despite volume resuscitation, requiring vasopressors to maintain MAP ≥65 mmHg | Hypotension: 1. systolic blood pressure <90 mmHg; 2. MAP < 65 mmHg; 3. a drop of > 40 mmHg of systolic blood pressure from baseline |
| (2) Serum lactate level > ULN† and using vasoactive drug (excluding single rescue medication) | + |

SBP <90 mm Hg, or mean arterial pressure <65 mm Hg, or SBP decrease >40 mm Hg from baseline, or serum lactate >2.0 mmol/L

*The laboratory RT-PCR procedures and cycle threshold (Ct) denoted positive findings varied from suppliers. PCR positive defined as a Ct of less than indicated threshold in each instruction.

†ULN, upper limit of normal. The ULN was defined according to the criteria by the laboratory standards in each hospital.

**Online Table II** The normal range of serum CRP, procalcitonin, creatinine, D-dimer, and platelet test in study hospitals

| | Normal Range for Serum Test | | | | |
|---|---|---|---|---|---|
| | **CRP** | **Procalcitonin** | **Creatinine** | **D-dimer** | **Platelets** |
| Site 1 | 0-10(mg/L) | 0-0.05(ng/ml) | 64-104(umol/L) | 0-500(ng/ml) | 0.50-2.50(mmol/L) |
| Site 2 | 0-10(mg/L) | 0-0.10(ng/ml) | 41-81(umol/L) | 0-0.55(mg/L) | 0.50-1.50(mmol/L) |
| Site 3 | 0-5(mg/L) | 0-5.00(ug/L) | 53-106(umol/L) | 0-0.55(mg/L) /0-1(mg/L) | 1.65-2.90(mmol/L) |
| Site 4 | 0-5(mg/L) | 0-0.05(ng/ml) | 40-105(umol/L) | 0-0.50(mg/L) | 1.65-2.90(mmol/L) |
| Site 5 | 0-10(mg/L) | 0-0.10(ng/ml) | 57-97(umol/L) | 0-0.24(mg/L) | 0.50-2.20(mmol/L) |
| Site 6 | 0-3(mg/L) | 0-0.50(ng/mL) | 57-111(umol/L) | 0-1(ug/mL) | 0.50-2.20(mmol/L) |
| Site 7 | 0-4(mg/L) | 0-0.05(ng/ml) | 64-104(umol/L) | 0-0.55(mg/L) | 0.50-2.50(mmol/L) |
| Site 8 | 0-10(mg/L)/0-5(mg/L) | 0-0.10(ng/ml) /0-0.50(ng/mL) | 38-120(umol/L) | 0-243(ng/ml) | 0.00-3.00(mmol/L) |
| Site 9 | 0-10(mg/L) | 0-0.50(ng/mL) | 35-110(umol/L) | 0-0.24(ug/ml) | 0.50-2.20(mmol/L) |

Abbreviations: CRP, C-reactive protein. Site 1, Zhongnan Hospital of Wuhan University; Site 2, Renmin Hospital of Wuhan University; Site 3, Wuhan First Hospital; Site 4, Wuhan Third Hospital; Site 5, Wuhan Seventh Hospital; Site 6, Wuhan Ninth Hospital; Site 7, Thunder Mountain Hospital; Site 8, Huanggang Central Hospital; Site 9, Central Hospital of Enshi Tujia and Miao Autonomous Prefecture.

**Online Table III** Clinical characteristics, laboratory data and CT features of patients on admission

| Parameters | Total (n=3430) | Hypertension† (n=1128) | Non-hypertension‡ (n=2302) | *P* value§ |
|---|---|---|---|---|
| **Clinical characteristics** | | | | |
| Age, median(IQR), y | 57(45-65) | 64(56-69) | 52(40-62) | 1.40E-107 |
| Male gender, n (%) | 1675(48.8) | 603(53.5) | 1072(46.6) | 1.73E-4 |
| Female gender, n (%) | 1755(51.2) | 525(46.5) | 1230(53.4) | 1.73E-4 |
| Heart rate, median(IQR), bpm | 82(76-93) | 84(78-96) | 81(76-92) | 5.21E-4 |
| Respiratory rate, median(IQR) | 20(19-21) | 20(19-22) | 20(19-21) | 0.004 |
| SBP, median(IQR), mmHg | 126(119-136) | 132(120-145) | 124(117-132) | 1.70E-42 |
| DBP, median(IQR), mmHg | 78(70-83) | 80(72-87) | 77(70-81) | 2.06E-15 |
| Days from symptom onset to hospitalization, median(IQR) | 10(7-15) | 10(7-15) | 10(6-14) | 1.26E-4 |
| Fever, n(%) | 2534(73.9) | 826(73.2) | 1708(74.2) | 0.57 |
| Cough, n(%) | 2331(68.0) | 775(68.7) | 1556(67.6) | 0.54 |
| Fatigue, n(%) | 1228(35.8) | 428(37.9) | 800(34.8) | 0.07 |
| Dyspnea, n(%) | 719(21.0) | 298(26.4) | 421(18.3) | 5.01E-8 |
| **Comorbidities** | | | | |
| Diabetes, n(%) | 388(11.3) | 240(21.3) | 148(6.4) | 9.76E-38 |
| Coronary heart disease, n(%) | 178(5.2) | 131(11.6) | 47(2.0) | 4.34E-32 |
| Chronic liver disease, n(%) | 62(1.8) | 21(1.9) | 41(1.8) | 0.98 |
| Cerebrovascular diseases, n(%) | 50(1.5) | 41(3.6) | 9(0.4) | 2.99E-13 |
| Chronic renal diseases, n(%) | 52(1.5) | 35(3.1) | 17(0.7) | 2.28E-7 |
| COPD, n(%) | 19(0.6) | 6(0.5) | 13(0.6) | 1.00 |
| **Chest CT** | | | | |
| Unilateral lesion, n/N(%) | 326/3216(10.1) | 58/1068(5.4) | 268/2148(12.5) | 6.70E-10 |
| Bilateral lesions, n/N (%) | 2721/3216(84.6) | 941/1068(88.1) | 1780/2148(82.9) | 1.30E-4 |
| **Laboratory examination** | | | | |
| Leukocytecount>9.5, 10^9/L, n/N (%) | 267/3191(8.4) | 123/1066(11.5) | 144/2125(6.8) | 6.35E-6 |
| Neutrophil count > 6.3, 9.5 10^9/L, n/N (%) | 442/3186(13.9) | 211/1065(19.8) | 231/2121(10.9) | 9.25E-12 |
| Lymphocyte count < 1.1, 9.5 10^9/L, n/N (%) | 1361/3187(42.7) | 508/1066(47.7) | 853/2121(40.2) | 7.27E-5 |
| C-reactive protein increase>ULN*, n/N (%) | 1361/2226(61.1) | 521/774(67.3) | 840/1452(57.9) | 1.59E-5 |
| Procalcitonin level increase> ULN*, n/N (%) | 633/2503(25.3) | 277/871(31.8) | 356/1632(21.8) | 5.70E-8 |
| ALT increase> 40 U/L, n/N (%) | 555/3141(17.7) | 203/1056(19.2) | 352/2085(16.9) | 0.12 |
| AST increase> 40 U/L, n/N (%) | 718/3144(22.8) | 287/1056(27.2) | 431/2088(20.6) | 4.53E-5 |
| Creatinine>ULN*, n/N (%) | 178/3009(5.9) | 106/1011(10.5) | 72/1998(3.6) | 7.70E-14 |
| D-dimer> ULN*, n/N (%) | 1055/2567(41.1) | 459/892(51.4) | 596/1675(35.6) | 9.77E-15 |
| Platelets < ULN, n/N (%) | 316/3192(9.21) | 188/2126(8.5) | 128/1066(12.0) | 0.005 |
| $K^+$ < 3.5 mmol/L, n/N (%) | 690/3132(22.0) | 284/1052(27.0) | 406/2080(19.5) | 2.33E-6 |
| LDL-c, mmol/L, median (IQR) | 2.3 (1.9-2.8) [n=2311] | 2.3 (1.8-2.8) [n=872] | 2.3(1.9-2.8) [n=1439] | 0.10 |
| SaO2, <95%, n/N (%) | 398/2442(16.3) | 176/830(21.2) | 222/1612(13.8) | 3.27E-6 |
| Blood glucose, median (IQR), mmol/L | 5.7(4.9-7.3) [n = 2797] | 6.2(5.2-8.3) [n = 966] | 5.5(4.8-6.8) [n = 1831] | 8.89E-24 |

Abbreviations: SBP, Systolic blood pressure; DBP, Diastolic blood pressure; COPD, Chronic obstructive pulmonary disease; ALT, alanine transaminase; AST, Aspartate transaminase; IQR, Interquartile range.

[*] Upper limit of normal (ULN) was defined according to criteria in each hospital and normal ranges of tests in each hospital were provided in Online Table 2.

[†] 1128 participants with a history of hypertension enrolled in the hypertension cohort.

[‡] 2302 participants without a history of hypertension enrolled in the non-hypertension cohort.

[§] The *P* values were calculated by Mann-Whitney U test for continuous variables, while categorical variables were compared by Fisher's exact test or Chi-square test between Hypertension and Non-hypertension group.

**Online Table IV** Characteristics of ACEI/ARB and non-ACEI/ARB groups among patients with hypertension taking antihypertensive drugs before and after propensity score-matching.

| | Unmatched | | | Matched [a] | | |
|---|---|---|---|---|---|---|
| **Parameters** | **ACEI/ARB[†] (n = 188)** | **Non-ACEI/ARB[‡] (n = 557)** | **SD[§]** | **ACEI/ARB[†] (n = 181)** | **Non-ACEI/ARB[‡] (n = 181)** | **SD[§]** |
| **Clinical characteristics on admission** | | | | | | |
| Age, median(IQR) | 64 (55-68) | 64 (57-68) | -0.061 | 64(56-68) | 64(57-69) | -0.056 |
| Male gender, n (%) | 100(53.2) | 277(49.7) | 0.069 | 95(52.5) | 92(50.8) | 0.033 |
| Female gender, n (%) | 88(46.8) | 280(50.3) | -0.069 | 86(47.5) | 89(49.2) | -0.033 |
| Heart rate, median(IQR) | 82.0(76.0-95.3) | 82.0(78.0-94.8) | -0.014 | 82(76.3-95.0) | 80(76.3-94.0) | 0.036 |
| Respiratory rate, median(IQR) | 20.0(19.0-21.0) | 20.0(19.0-22.0) | -0.022 | 20(19.0-21.0) | 20(19.0-21.8) | 0.081 |
| SBP, median(IQR) | 132.5(123.0-145.8) | 134.0(121.0-145.0) | 0.019 | 133.5(124.0-147.3) | 132(120.0-145.0) | 0.045 |
| DBP, median(IQR) | 80.0(72.0-87.8) | 80.0(73.0-86.0) | -0.010 | 80(72.0-88.0) | 80(73.8-88.0) | -0.025 |
| Symptom onset to admission, median(IQR), day | 10.0(7.0-15.0) | 10.0(7.0-15.0) | -0.078 | 10(7-15) | 9.5(6-15) | -0.031 |
| Fever, n(%) | 126(67.0) | 411(73.8) | -0.149 | 125(69.1) | 119(65.8) | 0.071 |
| Cough, n(%) | 122(64.9) | 379(68.0) | -0.067 | 118(65.2) | 113(62.4) | 0.058 |
| Fatigue, n(%) | 65(34.6) | 216(38.8) | -0.087 | 62(34.3) | 70(38.7) | -0.092 |
| Dyspnea, n(%) | 38(20.2) | 148(26.6) | -0.151 | 37(20.4) | 38(21.0) | -0.014 |
| **Comorbidities on admission** | | | | | | |
| Diabetes, n(%) | 44(23.4) | 118(21.2) | 0.053 | 42(23.2) | 42(23.2) | 0.000 |
| Coronary heart disease, n(%) | 29(15.4) | 56(10.1) | 0.162 | 25(13.8) | 28(15.5) | -0.047 |
| Chronic renal diseases, n(%) | 7(3.7) | 19(3.4) | 0.017 | 7(3.9) | 8(4.4) | -0.028 |
| Cerebrovascular diseases, n(%) | 5(2.7) | 18(3.2) | -0.034 | 5(2.8) | 5(2.8) | 0.000 |
| Chronic liver disease, n(%) | 4(2.1) | 7(1.3) | 0.068 | 4(2.2) | 4(2.2) | 0.000 |
| Chronic obstructive pulmonary disease, n(%) | 1(0.5) | 3(0.5) | -0.001 | 1(0.6) | 1(0.6) | 0.000 |
| **Chest CT on admission** | | | | | | |
| Unilateral lesion, n/N(%) | 16/173(9.3) | 27/525(5.1) | 0.159 | 14(8.4) | 12(7.1) | 0.050 |
| Bilateral lesions, n/N (%) | 146/173(84.4) | 474/525(90.3) | -0.178 | 143(86.1) | 150(88.8) | -0.079 |
| **Laboratory examination on admission** | | | | | | |
| Leukocyte count > 9.5, 10^9/L, n/N (%) | 22/183(12.0) | 55/527(10.4) | 0.050 | 22/177(12.4) | 17/175(9.7) | 0.087 |
| Neutrophil count > 6.3, 9.5 10^9/L ,n/N (%) | 32/183(17.5) | 100/527(19.0) | -0.039 | 32/177(18.1) | 38/175(21.7) | -0.091 |
| Lymphocyte count <1.1, 9.5 10^9/L, n/N (%) | 82/183(44.8) | 228/527(43.3) | 0.031 | 81/177(45.8) | 77/175(44.0) | 0.035 |

| | Unmatched | | | Matched [a] | | |
|---|---|---|---|---|---|---|
| **Parameters** | **ACEI/ARB[†] (N = 188)** | **Non-ACEI/ARB[‡] (N = 557)** | **SD** | **ACEI/ARB[†] (N = 181)** | **Non-ACEI/ARB[‡] (N = 181)** | **SD** |
| C-reactive protein increase>ULN[*], n/N (%) | 78/133(58.7) | 242/374(64.7) | -0.125 | 78/129(60.5) | 70/119(58.8) | 0.033 |
| Procalcitonin level increase> ULN[*], n/N (%) | 40/144(27.8) | 135/432(31.3) | -0.076 | 39/140(27.9) | 48/142(33.8) | -0.129 |
| ALT increase> 40 U/L, n/N (%) | 33/179(18.4) | 100/523(19.1) | -0.018 | 32/173(18.5) | 31/173(17.9) | 0.015 |
| AST increase> 40 U/L, n/N (%) | 40/179(22.4) | 133/523(25.4) | -0.072 | 39/173(22.5) | 41/173(23.7) | -0.027 |
| Creatinine>ULN[a], n/N (%) | 14/166(8.4) | 49/504(9.7) | -0.045 | 14/160(8.8) | 19/166(11.5) | -0.090 |
| D-dimer> ULN[a], n/N (%) | 67/149(45.0) | 240/445(53.9) | -0.180 | 67/144(46.5) | 76/145(52.4) | -0.118 |
| $K^+$ < 3.5 mmol/L, n/N (%) | 51/179(28.5) | 135/522(25.9) | 0.059 | 50/173(28.9) | 45/174(25.9) | 0.068 |
| Platelets < ULN, n/N (%) | 22/183(12.02) | 50/477(9.49) | 0.058 | 22/177(12.43) | 18/175(10.29) | 0.048 |
| LDL-c, mmol/L, median (IQR) | 2.2(1.8-2.8) [n = 151] | 2.3(1.9-2.8) [n = 426] | -0.069 | 2.23(1.8-2.8) [n = 146] | 2.34(1.8-2.8) [n = 142] | 0.011 |
| SaO2, <95%, n/N (%) | 30/138(21.7) | 84/413(20.3) | 0.034 | 29/132(22.0) | 28/134(20.9) | 0.026 |
| Blood glucose, median (IQR), mmol/L | 6.0(5.1-8.3) [n = 163] | 6.2(5.2-8.2) [n = 483] | 0.028 | 6.05(5.1-8.3) [n = 158] | 6.14(5.2-8.2) [n = 163] | 0.036 |

Abbreviations: ACEI, angiotensin converting enzyme inhibitor; ARB, angiotensin II receptor blocker; SBP, Systolic blood pressure; DBP, Diastolic blood pressure; COPD, Chronic obstructive pulmonary disease; ALT, alanine transaminase; AST, Aspartate transaminase; IQR, Interquartile range.

[*]Upper limit of normal (ULN) was defined according to criteria in each hospital and normal ranges of tests in each hospital were provided in Online Table 2.

[†]Patients with hypertension who taking ACEI and/or ARB during hospitalization were enrolled in the ACEI/ARB cohort. Patients discontinued treatment of hypertension due to inability to take medications or hypotension were not excluded from the cohort.

[‡]Patients with antihypertension drug who never taking ACEI and ARB during hospitalization were enrolled in the non-ACEI/ARB cohort.

[§]SD, Standardized differences be used to compare the mean of baseline covariate between ACEI/ARB and non-ACEI/ARB group.

**Online Table V** Hazard ratio and incidence rate ratios for association between ACEI/ARB and COVID-19 outcomes among patients with hypertension taking antihypertensive drugs in mixed-effect Cox model after propensity score-matching

| | **Mixed-effect Cox Model After PSM in patients taking antihypertensive drugs (1:2)** [*] | | |
|---|---|---|---|
| **ACEI/ARB vs non-ACEI/ARB** | **IRD** | **HR(95%CI)** | ***P* value**[†] |
| All-cause mortality | -0.36(-0.60,-0.12) | 0.29(0.12,0.69) | 0.005 |
| Septic shock | -0.31(-0.54,-0.09) | 0.24(0.10,0.63) | 0.003 |
| ARDS | -0.27(-0.64,0.10) | 0.73(0.44,1.19) | 0.21 |
| DIC | -0.14(-0.25,-0.03) | -[‡] | -[‡] |
| Acute kidney injury | -0.06(-0.28,0.16) | 0.83(0.36,1.87) | 0.65 |
| Acute heart injury | -0.16(-0.47,0.14) | 0.81(0.44,1.47) | 0.49 |

Abbreviations: HR, Hazard ratio; CI, Confidence interval; IRD, incidence rate differences, an absolute difference in the incidence between two groups.

[*] In the mixed-effect Cox model after propensity score matching, age, gender, cough, dyspnea, comorbidities (diabetes, coronary heart disease and chronic renal disease), CT-diagnosed lung lesions, and incidence of increased CRP and creatine were matched. Modeled center as a random effect in the multivariate analyses followed by adjustment for antiviral drug and lipid lowering drug.

[†] The *P* values were calculated by Fisher's exact test or $\chi 2$ test.

[‡] The incidence of DIC was 0 in the ACEI/ARB cohort. HR in DIC was not calculated.

**Online Table VI** In-hospital management of participants

| Management | Total (N=3430) | Hypertension* (n=1128) | Non-hypertension† (n=2302) | *P* value‖ |
|---|---|---|---|---|
| Traditional Chinese medicine% | 2921(85.2) | 978(86.7) | 1943(84.4) | 0.08 |
| Antiviral drug, n (%) | 2872(83.7) | 935(82.9) | 1937(84.1) | 0.38 |
| Antibiotics drug, n (%) | 2524(73.6) | 862(76.4) | 1662(72.2) | 0.01 |
| Nasal cannula Oxygen inhalation‡, n (%) | 2436(71.0) | 887(78.6) | 1549(67.3) | 7.87E-12 |
| Systemic corticosteroids, n (%) | 1185(34.6) | 446(39.5) | 739(32.1) | 2.00E-5 |
| Immunoglobin, n (%) | 918(26.8) | 330(29.3) | 588(25.5) | 0.02 |
| Antidiabetic drug, n (%) | 486(14.2) | 281(24.9) | 205(8.91) | 2.85E-36 |
| Lipid lowering drug, n (%) | 214(6.2) | 137(12.2) | 77(3.3) | 2.89E-23 |
| Vasoactive drug, n (%) | 220(6.4) | 124(11.0) | 96(4.2) | 3.25E-14 |
| Noninvasive ventilation§, n (%) | 206(6.0) | 122(10.8) | 84(3.7) | 1.99E-16 |
| Invasive ventilation§, n (%) | 96(2.8) | 60(5.3) | 36(1.6) | 7.54E-10 |
| Antifungal medications, n (%) | 81(2.4) | 47(4.2) | 34(1.5) | 1.99E-6 |
| Renal replacement therapy,n(%) | 19(0.6) | 15(1.3) | 4(0.2) | 5.33E-5 |
| Extracorporeal membrane oxygenation§, n (%) | 7(0.2) | 4(0.4) | 3(0.1) | 0.23 |
| Anti-hypertensive drug, n (%) | | | | |
| ACEI, n (%) | 31(0.9) | 31(2.8) | 0(0) | 6.29E-15 |
| ARB, n (%) | 157(4.6) | 157(13.9) | 0(0) | 2.62E-74 |
| CCB, n (%) | 592(17.3) | 592(52.5) | 0(0) | 1.12E-318 |
| Diuretic, n (%) | 300(8.8) | 178(15.8) | 122(5.3) | 3.57E-24 |
| beta-blocker, n (%) | 334(9.7) | 221(19.6) | 113(4.9) | 6.38E-42 |
| alpha-blocker, n (%) | 35(1.0) | 25(2.2) | 10(0.4) | 2.63E-6 |

Abbreviations: CCB, calcium channel blockers; ACEI, angiotensin converting enzyme inhibitor; ARB, angiotensin II receptor blocker.

*1128 participants with a history of hypertension enrolled in the hypertension cohort.

†2302 participants without a history of hypertension enrolled in the non-hypertension cohort.

‡Nasal cannula oxygen inhalation was taken in isolation.

§Noninvasive ventilation, invasive ventilation, and extracorporeal membrane oxygenation are at mutually exclusive.

‖ The *P* values were calculated by Mann-Whitney U test for continuous variables, while categorical variables were compared by Fisher's exact test or Chi-square test.

.

**Online Table VII** Hazard Ratio for primary and secondary outcome in hypertension and non-hypertension group

| | Unadjusted | | Adjusted[*] | |
|---|---|---|---|---|
| Hypertension vs non-hypertension | HR(95%CI) | *P* value[†] | HR(95%CI) | *P* value[†] |
| All-cause mortality | 2.612(1.95,3.51) | 0.000 | 1.41(1.03,1.94) | 0.03 |
| Septic shock | 3.51(2.47,5.01) | 0.000 | 1.95(1.33,2.85) | 0.001 |
| ARDS | 2.42(2.01,2.91) | 0.000 | 1.61(1.32,1.97) | 0.000 |
| DIC | 5.84(2.60,13.11) | 0.000 | 2.74(1.15,6.54) | 0.02 |
| Acute kidney injury | 4.08(2.62,6.34) | 0.43 | 2.55(1.58,4.12) | 0.000 |
| Acute heart injury | 3.05(2.34,3.96) | 0.000 | 1.68(1.26,2.23) | 0.000 |

Abbreviations: ARDS, Acute respiratory distress syndrome; DIC, disseminated intravascular coagulation; HR, Hazard ratio; CI, Confidence interval.

[*]The adjusted variables included age, gender, comorbid diabetes, cerebrovascular diseases, and chronic renal disease.

[†]The *P* values were calculated based on Cox proportional hazard model.

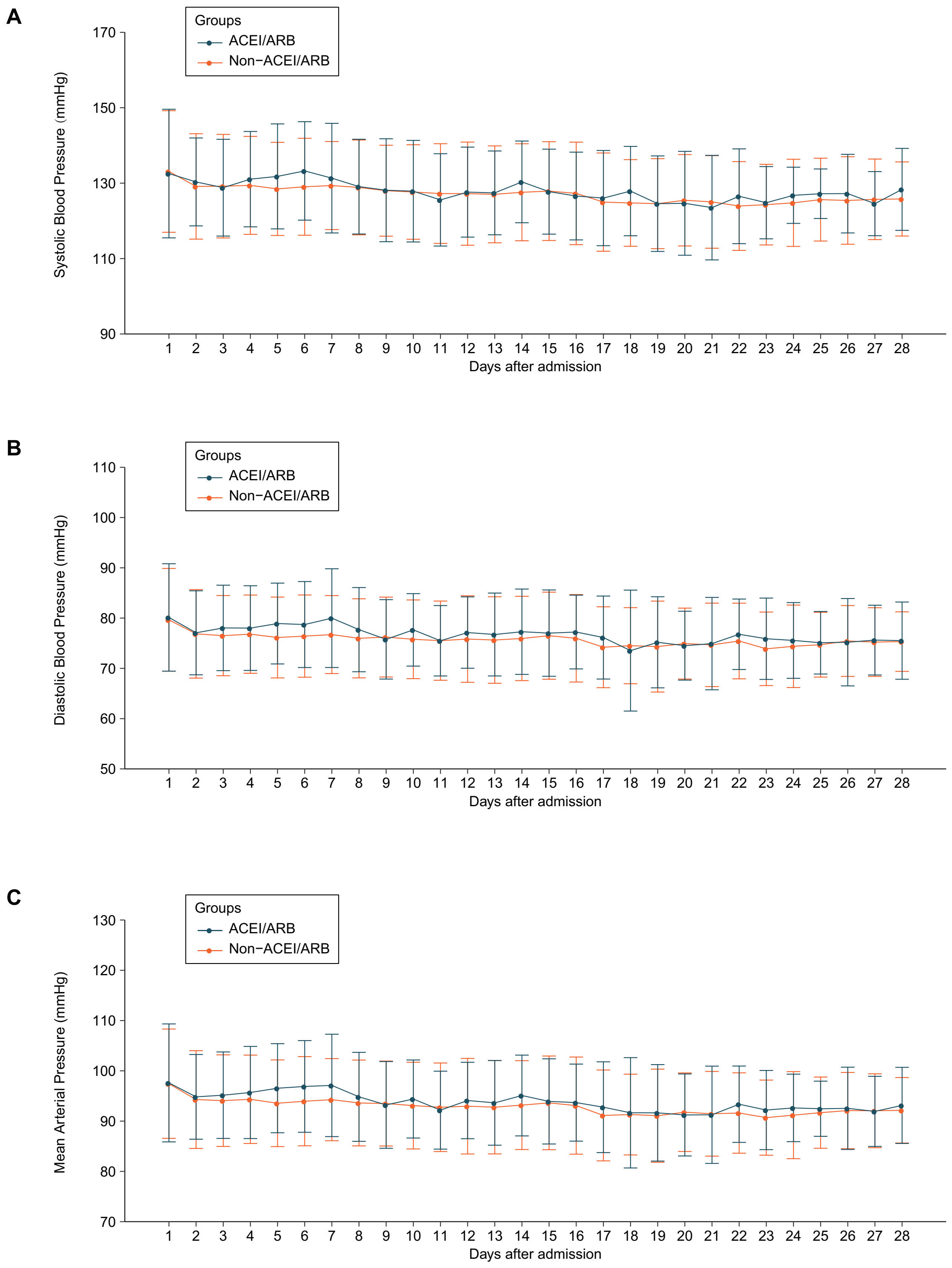


**Online Figure I** Dynamic change of blood pressure and mean arterial pressure of patients with hypertension treated with ACEI/ARB or without ACEI/ARB. (A-C) The systolic pressure (A), diastolic pressure (B), and mean arterial pressure (C) of patients with hypertension in a 28-day follow-up duration from admission.

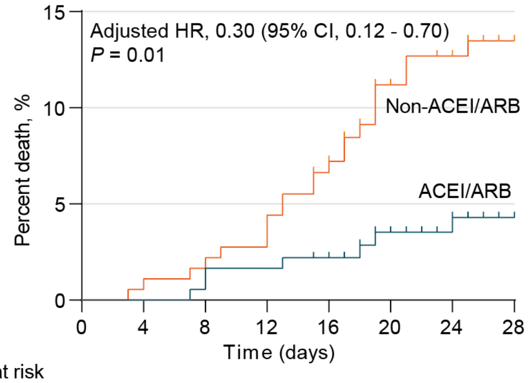


No. at risk

| | | | | | | | | |
|---|---|---|---|---|---|---|---|---|
| Non-ACEI/ARB | 181 | 180 | 178 | 176 | 158 | 121 | 114 | 106 |
| ACEI/ARB | 181 | 181 | 180 | 178 | 167 | 137 | 128 | 121 |

**Online Figure II.** Kaplan-Meier Curves for cumulative probability of COVID-19 mortality among individuals with inpatient use of ACEI/ARB (ACEI/ARB cohort) or taking other antihypertensive drugs (non-ACEI/ARB cohort) in propensity score-matched analysis. The blips indicate censoring. The median (IQR) observation time was 28 (20-28) in ACEI/ARB cohort and 28 (18-28) in non-ACEI/ARB cohort.

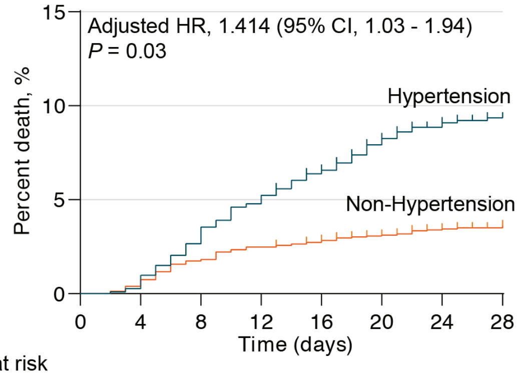


| No. at risk | | | | | | | | |
|---|---|---|---|---|---|---|---|---|
| Hypertension | 1148 | 1145 | 1116 | 1092 | 1019 | 820 | 749 | 689 |
| Non-Hypertension | 2307 | 2298 | 2267 | 2250 | 2124 | 1852 | 1760 | 1658 |

**Online Figure III.** The 28-day Kaplan-Meier Curves of hypertensive and non-hypertensive cohort. The blips indicate censoring. Patients with hypertensive status were identified according to their electronic medical record and disease history.
