# Supplementary figures and images for: Association of Inpatient Use of Angiotensin-Converting Enzyme Inhibitors and Angiotensin II Receptor Blockers With Mortality Among Patients With Hypertension Hospitalized With COVID-19
Source: Circ Res. 2020 Jun 2;126(12):1671–81. doi: 10.1161/CIRCRESAHA.120.317134 (PMC7265882; doi:10.1161/CIRCRESAHA.120.317134)

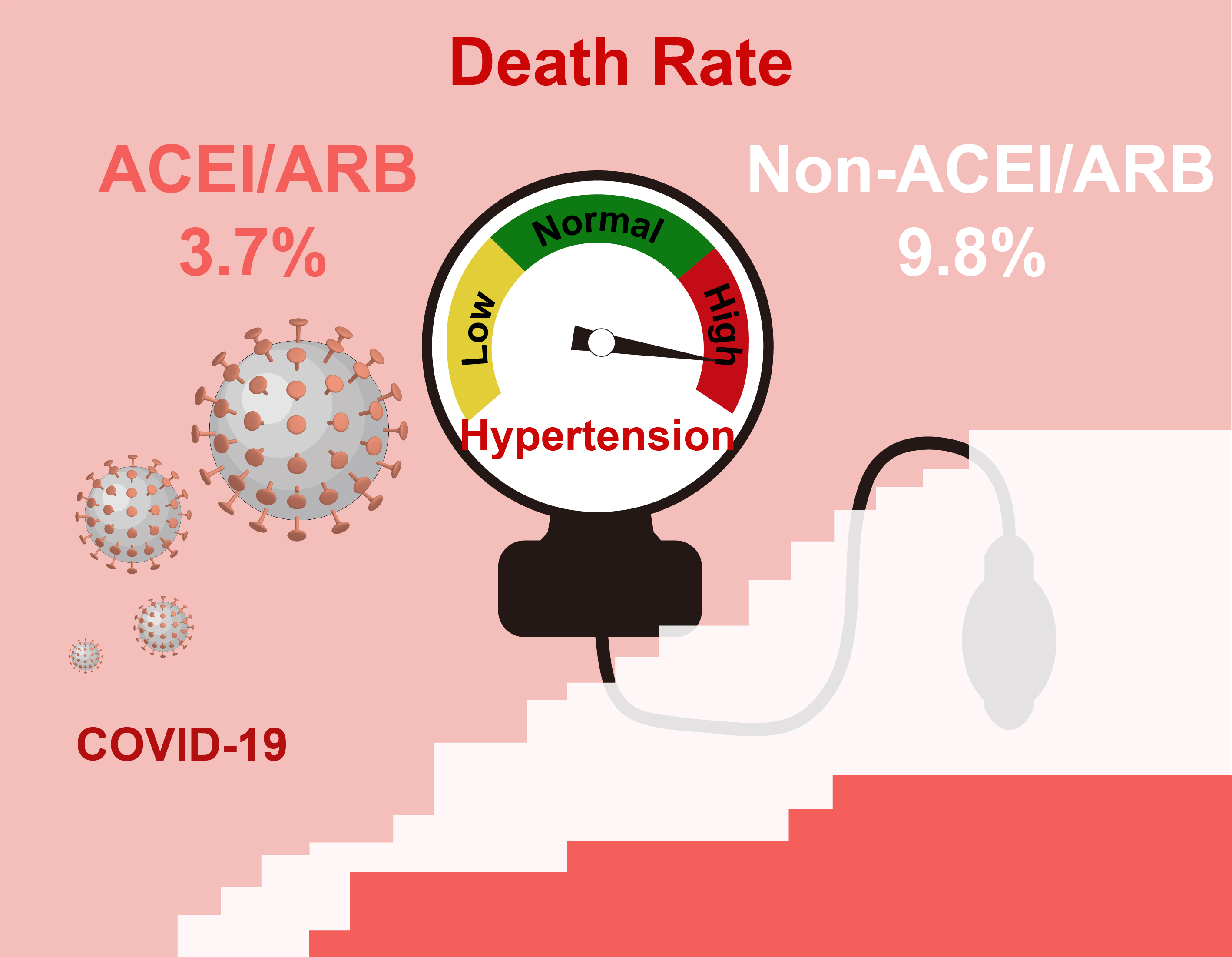

Supplement: Supplementary file 2 [file res-126-1671-s002.jpg]
